# Cereal and Confectionary Packaging: Assessment of Sustainability and Environmental Impact with a Special Focus on Greenhouse Gas Emissions

**DOI:** 10.3390/foods11091347

**Published:** 2022-05-06

**Authors:** Victoria Krauter, Anna-Sophia Bauer, Maria Milousi, Krisztina Rita Dörnyei, Greg Ganczewski, Kärt Leppik, Jan Krepil, Theodoros Varzakas

**Affiliations:** 1 Packaging and Resource Management, Department Applied Life Sciences, FH Campus Wien, University of Applied Sciences, 1030 Vienna, Austria; anna-sophia.bauer@fh-campuswien.ac.at (A.-S.B.); jan.krepil@fh-campuswien.ac.at (J.K.); 2 Department of Chemical Engineering, University of Western Macedonia, 50100 Kozani, Greece; mmilousi@uowm.gr; 3 Institute of Marketing, Corvinus University of Budapest, 1093 Budapest, Hungary; krisztina.dornyei@uni-corvinus.hu; 4 Management in Networked and Digital Societies (MINDS) Department, Kozminski University, 03-301 Warsaw, Poland; ganczewski@gmail.com; 5 Center of Food and Fermentation Technologies, 12618 Tallinn, Estonia; kart@tftak.eu; 6 Department of Chemistry and Biotechnology, School of Science, Tallinn University of Technology, 19086 Tallinn, Estonia; 7 Department of Food Science and Technology, University of Peloponnese, 24100 Kalamata, Greece; theovarzakas@yahoo.gr

**Keywords:** food, packaging, cereals, confectionary, snacks, life cycle assessment, LCA, environmental impact, CO_2_ footprint, food losses and food waste

## Abstract

The usefulness of food packaging is often questioned in the public debate about (ecological) sustainability. While worldwide packaging-related CO_2_ emissions are accountable for approximately 5% of emissions, specific packaging solutions can reach significantly higher values depending on use case and product group. Unlike other groups, greenhouse gas (GHG) emissions and life cycle assessment (LCA) of cereal and confectionary products have not been the focus of comprehensive reviews so far. Consequently, the present review first contextualizes packaging, sustainability and related LCA methods and then depicts how cereal and confectionary packaging has been presented in different LCA studies. The results reveal that only a few studies sufficiently include (primary, secondary and tertiary) packaging in LCAs and when they do, the focus is mainly on the direct (e.g., material used) rather than indirect environmental impacts (e.g., food losses and waste) of the like. In addition, it is shown that the packaging of cereals and confectionary contributes on average 9.18% to GHG emissions of the entire food packaging system. Finally, recommendations on how to improve packaging sustainability, how to better include packaging in LCAs and how to reflect this in management-related activities are displayed.

## 1. Introduction

The sustainability of food and, in particular, its packaging continues to be at the center of public and political debate. In order to make objective and knowledge-based decisions, it is of utmost importance to understand the requirements of a food product on its packaging on the one hand and to be able to select the optimal packaging solution for the respective purpose on the other hand. While the former has already been covered in the review paper “Cereal and Confectionary Packaging: Background, Application and Shelf-Life Extension” [1], the present review aims to address the important issue of sustainability and assessment thereof.

Recently, it has been shown and further substantiated by Crippa et al. that food systems are accountable for a major share, namely 34%, of global anthropogenic greenhouse gas (GHG) emissions (data representing 2015). The authors also showed that this percentage predominantly originates from agriculture and land-use and land-use change activities (71%). The remaining fraction (29%) represents activities along the food supply chain such as processing, distribution (e.g., packaging, retail, transport), consumption and corresponding end-of-life scenarios. Being of increased importance and use, packaging resulted in a 5.4% share, which was calculated considering relevant materials and industries (e.g., pulp and paper, aluminum, metal, glass). This value is slightly above the shares for transportation (4.8%) and the cold chain (5%) [2].

The seemingly relatively small contribution of packaging to total GHG emissions in relation to food products against the background of current discussions about packaging and sustainability has also been shown by Poore and Nemecek [3]. The authors likewise calculated a 5% share of packaging but also showed that the results for product groups differed greatly from one another. For instance, alcoholic beverages, such as beer and wine, exhibited packaging-related emissions of around 40% (with glass packaging as the main driving impact factor), while fruit and vegetables showed packaging-related emissions of around 10 to 20% [3]. This difference in the impact ratio between packaging and food for different products has also been shown by other authors and studies [4,5,6,7]. For example, Verghese et al. stated that packaging of meat, fish and eggs accounts for 2% of GHG emissions, while packaging for dairy as well as fruits, vegetables and nuts account for 10 and 12%, respectively [6]. Heller et al. underlined this by visualizing that resource- and emission-intensive food products, such as meat or milk, tend to have a high food-to-packaging ratio, while less resource- and emission-intensive food products, such as leafy greens, show a small ratio [7].

Especially for food products with a (very) high impact, these results point out the importance of the protective function of packaging [6,7,8,9,10]. Optimizing and sometimes increasing packaging can reduce food losses and waste along the food supply chain while at the same time reducing the overall environmental impact [11]. For food products with a low impact, on the other hand, more precise consideration must be given to which packaging (e.g., material) should be used and which trade-offs must be considered [10,11,12,13,14]. Therefore, the sustainability (including ecological, economic and social dimensions) of product packaging systems is the subject of current research and finds more and more attention in policies and legislation [15,16,17].

Due to the great importance of high-impact foods (e.g., products of animal origin such as meat and milk [18]) and foods with high food losses and waste (e.g., fruits and vegetables), publications on these topics are a priority in the scientific literature. This is reflected by different studies and reviews [3,18,19,20,21,22]. However, to the author’s best knowledge, no comprehensive work taking into account the important group of cereal and confectionary products [23,24,25], their packaging and related GHG emissions exists. This shortcoming is also underlined by different authors [26,27,28,29,30,31,32]. Against this background, the aim of the present review is to:

- Contextualize packaging and sustainability as well as sustainability assessment methods;
- Display and discuss how and to what extent food packaging is included in existing life cycle assessments (LCAs) in the cereals and confectionary sector;
- Point out the environmental impact of cereal and confectionary packaging in relation to the food product with a special focus on GHG emissions;
- Highlight improvement strategies to optimize (cereal and confectionary) packaging systems as well as LCA of the same.

This provides a valuable basis for decision makers as well as practitioners in research, development and innovation to take further steps towards sustainable food packaging.

## 2. Packaging and Sustainability

### 2.1. Sustainable Packaging

#### 2.1.1. Definition

Despite its common usage, the term “sustainable packaging” is defined and utilized in different ways by various stakeholders along the food supply chain and beyond [33]. Accordingly, several approaches, frameworks and methodologies with differing foci, principles, criteria and connected indicators can be found in the relevant literature [34]. These, amongst others, encompass legal texts on packaging and packaging waste [35,36], guidelines for producers and retail focusing on specific topics such as design for recycling [37,38,39,40,41], as well as more holistic packaging sustainability frameworks [42,43,44,45].

A condensed but comprehensive framework is that of the Sustainable Packaging Alliance (Australia) [42]. This so-called Packaging Sustainability Framework defines a total of four principles, namely that sustainable packaging must be (i) effective, (ii) efficient, (iii) cyclic and (iv) safe. In this context, “effective” means that the respective packaging is fit for purpose and fulfils its essential functions (e.g., containment, protection, communication, convenience [46,47,48]) with as little effort as possible. “Efficient”, on the other hand, refers to packaging that minimizes resource consumption (e.g., materials) as well as emissions (e.g., CO_2_) along its life cycle and “cyclic” emphasizes that it is necessary to keep resources in the biological (e.g., bio-based or biodegradable materials) or technical (e.g., recycling, use of recycled materials) cycle. Furthermore, “safe” focuses on packaging that does not pose a risk to people (e.g., migration of harmful substances from the packaging material to the food product) or the environment (e.g., pollution) along its life cycle [42,43,45,49].

It is important to point out that the above four principles are closely interrelated and that (increased) efforts in one area can lead to positive or negative changes in another [43]. The latter case and corresponding trade-offs are represented, for example, by the use of multilayer flexible food packaging. While this often offers a high level of product protection (e.g., barrier) with low material input and correspondingly low emissions (e.g., CO_2_), the combination of different materials (e.g., different plastics, aluminium, paper) makes it difficult to recycle them [50]. Another possible trade-off is the reduction or minimization of packaging. While this is desirable in principle, underpackaging can lead to undermining the effectiveness of a packaging system, resulting in increased food losses and/or waste and corresponding environmental impacts. Overpackaging, on the other hand, also leads to elevated environmental impacts due to the excess material used [43].

#### 2.1.2. Development

Taking this into account, finding the optimum point (as little as possible, as much as necessary) with balancing the above-mentioned principles is of the utmost interest in a packaging (re)-design process. Since “THE” sustainable packaging is not a specific, existing product that can be applied to any given (food) product, but rather a system that must be constantly adapted to the changing needs of, for example, the (food) product, the value chain, consumers and legal requirements, the resulting “sustainable” packaging solutions can be as diverse as the initial factors [43].

Consequently, developing a successful packaging solution not only at the primary but also at the secondary and tertiary packaging level [51] is a complex and critical undertaking that requires dedication, investment and, most importantly, a holistic and collaborative approach [43,48,52]. While holistic refers to life cycle thinking and assessment, collaborative refers to pro-active and dedicated action of not only single actors but connected and communicating companies, supply chains, science and research as well as stakeholders such as governments or consumers. This allows the development of (eco)efficient and effective solutions that enable the transition from a linear to a circular economy and show benefits in multiple dimensions (ecologic, economic, social) [43,52,53,54,55,56].

**Figure 1:**
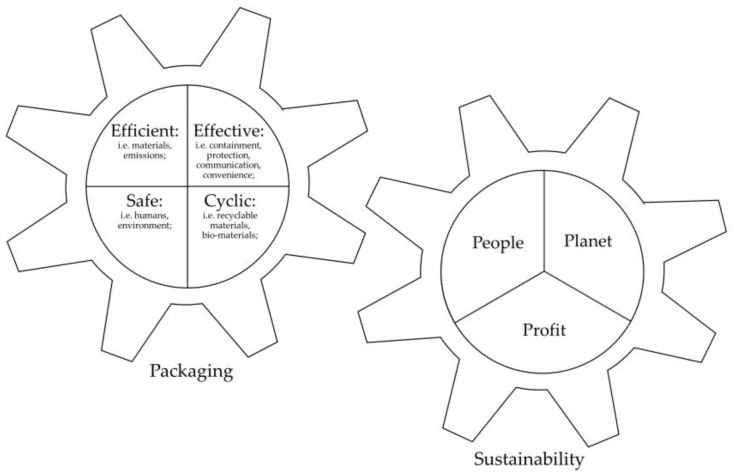
Principles of sustainable packaging and their impact on ecological, economic and social sustainability. Graphic based on [42,43,46].

To evaluate or compare different developed packaging solutions with regard to ecological, economic and social aspects, different criteria, indicators, metrics and evaluation methods can be used. While economic and social effects can be assessed using, for instance, Life Cycle Costing (LCC) [57,58,59,60,61] and Social Life Cycle Assessment [62,63,64,65], ecological effects are usually assessed using a (full) Life Cycle Assessment (LCA) ([66,67,68,69], simplified (or streamlined) LCA, non-LCA tools or scorecards (see also Figure 1) [70,71,72].

#### 2.1.3. Challenges

Sustainable packaging development frequently involves high production costs, long development time and technical difficulties [43,54]. Therefore, many sustainable packaging solutions are not implemented without significant sales increase or cost reduction. Findings also show that sustainable packaging ambitions often stay on the firm’s strategic level because companies might prioritize a product’s market potential and a limitation of commercial risks over sustainability considerations on an operational level. As a result, sustainable advances in packaging development frequently remain limited [73].

Companies’ sustainability commitment is also reduced if such packaging solutions’ commercial success is questionable or if it does not positively influence consumer behavior [53]. Unfortunately, from the consumer perspective, sustainable packaging does not always refer to a truly sustainable solution but to a specific design, which evokes explicitly or implicitly the perception of sustainability via its structure and its visual and informational cues [74,75]. Moreover, consumer perception of sustainable packaging is controversial: some consumers have a generally positive attitude toward sustainable packaging [76,77], and others regard such packaging as an environmental villain due to the way the media have recently communicated about packages. However, in general, they have limited awareness, recognition and knowledge of the different sustainable functions (such as labels, materials, disposal processes, and manufacturing technologies) of such packaging solutions [78,79,80] and often focus their environmental concerns solely on the packaging’s end-of-life [56]. They also associate sustainable packages with certain risks (lower perceived quality, lower functionality, less attractiveness, perceived contamination), which leads to lower perceived functionality and lower willingness to purchase [76,81]. Consumers can also be easily deceived by packaging communication [82], and some even perceive sustainable claims as greenwashing, especially when these claims are not in line with their subjective sustainable packaging expectations [80,83]. It is, therefore, important to study and include consumer insights in sustainability packaging analysis and also include other necessary steps to avoid failures [43].

### 2.2. Life Cycle Assessment

One of the first LCAs focusing on food packaging was initiated by the Midwest Research Institute (MRI) for the Coca-Cola Company in 1969 [70,84,85,86]. In 1974, the same institute conducted a follow up of this study for the United States Environmental Protection Agency [87]. Similarly, Unilever has performed several LCA studies for various product groups such as margarine and ice cream in the late 1980s. Since then, and in the context of the need for more sustainable products and processes, numerous further studies have been conducted in this research field [85,86,87,88,89,90,91,92,93,94,95]. Building on this, LCA has also increasingly found its way into more than just industrial decision-making [96]. For instance, a comparative LCA study on different beverage packaging formed the basis of the political decision of the German Federal Ministry for the Environment with regard to the German deposit system on disposable packaging (single-use deposit) in the early 2000s. However, since conditions (e.g., legal framework, economy, inventory data) are not static but constantly adapting, the study was repeated recently and is again influencing policy-making [97,98]. Being just one example, it is expected that LCA will be more and more applied to improve policy- and decision-making in the future (e.g., waste management policies) since it offers transparent and valuable information about the actual sustainability of a product or process. However, a sound methodology and expert knowledge in conducting such analyses is a prerequisite to achieving meaningful output [99,100,101].

A full LCA should consider the following life cycle stages: raw material extraction and preprocessing (cradle), transportation of processed materials to the manufacturing site, production of components, assembly of the system, transportation to market (gate), use phase and end-of-life with transportations of the used equipment to the intended waste treatment plant, e.g., landfill (grave) or recycling/material recovery (back to cradle). An LCA study can be: (i) partial, referring to some phases of the product’s lifecycle, i.e., cradle-to-gate, (ii) semi-complete, including landfilling or partial recycling, i.e., cradle-to-grave or (iii) complete, employing all life time phases and including material upscaling aspects as described in the circular economy principles, i.e., cradle-to-cradle [34]. The Product Environmental Footprint (PEF) is a multi-criteria method for modelling the potential environmental performance of a product, and it can easily be inferred through the LCA results, especially in cradle-grave or cradle-cradle approaches [102,103].

According to the guidance provided by the International Standardization Organization (ISO) in ISO 14040 and ISO 14044, an LCA study is generally carried out by iterating four distinct phases [66,67]:

In the first step, i.e., Goal and Scope, the objectives of the study are defined to clarify the intended application and the reasons for the study, including the target audience. Scope, on the other hand, describes the product system, as well as the functional unit (FU) and the system’s boundaries. The selection of the FU is a basis for comparing similar products. Thus, a typical FU relates to the overall product function rather than focusing on a particular physical property, while it is normally time-bounded and can correlate the expected duration of use and desired quality under certain circumstances. The meaningful selection and definition of system boundaries is a crucial task as it determines the overall type of the LCA, i.e., whether it is a cradle-to-gate, a cradle-to-grave or a cradle-to-cradle approach [104].

During the second step, i.e., Life Cycle Inventory analysis (LCI), a comprehensive inventory of energy, materials and environmental inputs-outputs is created, identifying and quantifying all related data at every stage of the life cycle. The collection of data and determination of total emissions and resource use take place alongside a detailed definition of entailed production processes. All collected data are scaled based on the preset functional unit for the studied system. Lack of data availability and quality is a typical drawback and can usually refer to studies related to non-standardized procedures. Other inhibiting factors are geographic variations regarding the quality of raw materials and energy sources, production methods and relevant environmental impacts [105].

The next and third step, i.e., Life Cycle Impact Assessment (LCIA), is the phase of an LCA with particular respect to sustainability assessment. During the impact assessment, the potential environmental impacts associated with identified inputs and outputs are categorized into different categories. During LCIA, emissions and resource extractions are translated into a limited number of environmental impact scores by means of so-called characterization factors. There are two mainstream ways to derive these factors, i.e., at the midpoint and at the endpoint level. Midpoint indicators focus on single environmental problems, for example, climate change or acidification, while endpoint indicators present environmental impacts on three higher aggregation levels, i.e., (i) effect on human health, (ii) biodiversity and (iii) resource scarcity [106].

In the fourth step, i.e., Interpretation, the results of the inventory analysis and the impact assessment are interpreted and combined in order to make a more informed decision. During this phase, a comparison of the results with previous studies is made in order to determine whether they are aligned with the literature. Furthermore, a sensitivity analysis can be performed to validate the consistency of the findings. ISO standards provide a general framework of an iterative nature. Thus, if the outcomes of the impact assessment are incomplete for drawing conclusions, then the previous LCA steps must be repeated until the final results support the initial goals of the study [107].

As LCA is by default a holistic method that accounts for multiple environmental impact categories, carbon footprint analysis evaluates the GHG emissions generated by a product, activity, or process that contributes to global warming, and it is a subset of a complete LCA. Thus, it is always based on international standards such as ISO 14040/14044, ISO 14067, PAS 2050, and the GHG Product Life Cycle Standard [66,67,108,109].

One important aspect of applying LCA in food packaging is to quantify the inherent direct and indirect effects in order to assess the environmental sustainability of the sector. Direct effects of packaging include impacts from the production and end-of-life of the related materials. Additionally, indirect effects derive from life cycle losses and waste that occur in different phases of the food supply chain [110].

## 3. Sustainability of Cereal and Confectionary Packaging

### 3.1. Literature Analysis

To display and discuss how and to which extent packaging is present in existing LCA studies in the cereal and confectionary sector and to point out the environmental impact (focus on GHG emissions) of the packaging in relation to the respective food product, a literature search in different databases was conducted, similar to Molina-Besch et al. [111]. Firstly, and for the identification of relevant LCA studies, the keywords “Life Cycle Assessment” and “Carbon Footprint” were used. Secondly, to identify relevant food products, keywords given in the guidance document in Part E of Annex II of the regulation (EC) No 1333/2008 on food additives were used. (Sub)categories considered were: confectionary products (cocoa and chocolate products, other confectionaries including breath-freshening micro-sweets), cereals and cereal products (whole, broken or flaked grain, flours and other milled products, breakfast cereals, pasta, noodles, batters, pre-cooked or processed cereals), bakery wares (bread and rolls, fine bakery wares) as well as ready-to-eat savories and snacks (potato-, cereal-, flour- or starch-based snacks, processed nuts) [112]. The first keywords were combined with “or”. The second keywords were individually added using “and”. Articles written in English and published since 2009 were considered for review. Of these, relevant studies including food, packaging and related LCA results were analyzed in detail. Where results (on packaging) were included in graphics (e.g., bar chart) but not in numeric form, the online tool Web-Plot Digitizer was used to extract the data [113]. Further, for each study, the percentage of packaging-related GHG emissions was taken from the results or extracted (calculated) where necessary.

Based on the available data set, commonalities and differences between the studies were investigated in a multi-step approach based on ISO 14040 and 14044: (i) goal and scope, (ii) life cycle inventory, (iii) life cycle impact assessment and (iv) interpretation [66,67]. This stands in contrast to Molina-Besch et al., who focused primarily on (i) and (iv) [111]. Since the present review not only aims to highlight how packaging is included in the studies but also to point out improvement opportunities for packaging and assessment, the authors also focused on LCA methodology, represented by (ii) and (iii).

As it is well known that the direct comparison of results from different LCA studies (e.g., due to different goals and scope, data used, cut-offs) is difficult [111,114,115], the present study aims at rather comparing approaches, magnitudes and ranges than exact values.

### 3.2. Results

#### 3.2.1. Goal and Scope

##### Focus

In total, 28 LCA studies covering 108 products in the categories of confectionary, cereals and cereal products, bakery wares and ready-to-eat savories and snacks fulfilled the above-given criteria (see also Table 1). Within these studies, products from the confectionary category (total 42%) and especially the sub-category of cocoa and chocolate products were assessed most frequently (38%). On the contrary, the sub-category of other confectionaries, including breath-freshening micro-sweets, only resulted in a low number of entries (4%). Products covered were, for example, jelly and foam sweets as well as sugar and milk-based confectionary. This focus on cocoa and chocolate products may be due to the high economic relevance of cocoa [23,24] and is well in line with, for example, the findings of Miah et al. [26], who stated that diverse confectionary products are underrepresented in LCA studies and that chocolate products dominate the literature body.

A total of 24% of the products were located in the area of cereal and cereal products. On the forefront in the sub-category of whole, broken or flaked grain (8%) was rice. For the sub-category of flours and other milled products and starches (3%), oat, potato and wheat were represented. Further, the sub-category of breakfast cereals (4%) was covered by one known brand’s products as well as porridge. The sub-category of pasta (9%) included different products made from different raw materials. Interestingly, the category of bakery wares (30%) showed an elevated number of packaged products in the sub-categories of bread and rolls (e.g., (sliced) bread) (19%) as well as fine bakery wares (e.g., biscuits, cakes) (11%).

Last but not least, the category ready-to-eat savories and snacks only displayed one product example (5%), namely crisps, for the sub-category of potato-, cereal-, flour- or starch-based snacks (1%) and some examples for the sub-category of processed nuts (e.g., pistachio) (4%).

##### Aim

Analyzing the studies with regard to packaging, it quickly becomes clear that the focus (overall goal and scope) is mainly on the food products themselves. Molina-Besch et al. [111] name these types of studies *food LCAs*, whereas studies with a focus on the impact of the packaging system are called *packaging LCAs*. In total, 7 out of 28 studies explicitly mentioned packaging in one form or another in their aim. While some studies seem to mention packaging in passing, others go more into detail. For example, Boakye-Yiadom et al. [116] mentioned “environmental impacts associated with the production of a packaged chocolate”, Cimini et al. [117] included “pasta in 0.5 kg polypropylene (PP) bags” in their aim, and Volpe et al. [118] focused on “bags of” nuts. Büsser and Jungbluth [119], on the other hand, aimed at analyzing “the environmental performance of packaging with respect to its function within the life cycle of chocolate” and Espinoza-Orias et al. [120] included “… the influence on the carbon footprint of several parameters … including … type of packaging (plastic and paper bags) …”. Further, with an explicit focus not only on the direct but also indirect effects of packaging, Svanes et al. [121] aimed to “… establish environmental hotspots; to examine the role of … packaging … and to identify potential measures to reduce this wastage”, and Williams and Wikström [11] aimed to “… analyze the potential of decreasing environmental impact of five food items … through the development of packaging that reduces food losses in the consumer phase”. These studies are, however, exceptions and mirror the findings of Molina-Besch et al. [111], who likewise, but for a wider product range, found that packaging is currently insufficiently considered in LCAs.

##### Functional Unit

The strong focus on the food product itself is also reflected by the functional units given; slightly more than half of the authors do not even name packaging in this regard [27,30,118,120,121,122,123,124,125,126,127,128,129,130,131]. Those who do [11,26,28,29,31,32,116,117,119,132,133,134] almost exclusively (with the exception of (Nilsson et al. [132]) give the functional unit as “one kilogram of product in the respective packaging”. This corresponds to a formulation as laid down in the Product Category Rules (PCR) rules of the International Environmental Product Declaration (EPD) system [31,135,136], as well as other sources [104,137].

In this context, EPDs, as such, which are based on LCAs, should also be discussed in a short excurse. According to the definition of ISO 14025, these are so-called Type III environmental declarations. Specifically, they are independently verified and registered documents that make the environmental impact of products transparent and comparable over their entire life cycle. Type I and II stand for third-party and self-declared eco-labels, respectively [138,139]. Interestingly, the EPD Library (search criteria: product category food & beverages; PCR bakery products) already contains more than 100 EPDs [140]. These are highly relevant for the present review with regard to the categories of cereals and cereal products as well as bakery wares, but outside the scope (e.g., scientific literature) defined in chapter 3.1. Moreover, the EPDs are structured very similarly to each other. Accordingly, these will not be analyzed in detail in the coming chapters but will be used for comparison and discussion where appropriate.

##### System/Scope

While a considerable amount of the studies reviewed followed a cradle-to-gate or a gate-to-gate approach [116,118,119,122,123,125,127,131,132,133,141], the majority considered the product life cycle in a cradle-to-grave approach [11,26,27,28,29,30,31,32,117,120,121,124,126,128,129,130,134]. The latter is a prerequisite for assessing not only the direct environmental effects of packaging (impacts caused by production and end-of-life) but also the indirect environmental effects of the same (influence on, e.g., food waste and transport efficiency), a research field gaining more and more importance due to the high environmental impacts of food systems and the valuable role of packaging in avoiding or reducing food losses and waste [19,43,111,142,143]. The packaging-relevant direct and indirect effects in this context are: primary packaging (direct), secondary and tertiary packaging (direct), transport from producer to retail (indirect), food waste in transport, distribution and retail (indirect), food transport, storage and preparation by households (indirect), food waste in households (indirect), packaging end-of-life (direct) and food waste end-of-life (indirect) [111].

On closer examination of the studies with a cradle-to-grave approach, it becomes apparent that some did not include all key LCA steps necessary to evaluate the indirect effects of packaging at the point of sale or consumption. Transport (from producer to retail as well as to households), however, was covered in almost all the studies in the form of distance travelled. Factors influenced by the packaging, such as transport efficiency due to efficient and/or lighter packaging, on the other hand, were not in the foreground [11,26,27,28,29,30,31,32,117,120,121,124,126,128,129,130,134]. Regarding food losses and waste during transport, distribution and retail, Miah et al. [26], for example, gave information on the percentage of waste generated at the different life cycle stages for confectionary. Likewise, Sieti et al. [130] did the same for breakfast cereals. Cimini et al. [117] even named package breakage as a reason for waste during distribution. Additionally, Svanes et al. [121] explicitly calculated the direct and indirect effects of waste at the production, retail and household level for bread and rolls. Further, information on food waste was included by Espinoza-Orias et al. [120] for bread and rolls, Konstantas [29] for cakes, Miah et al. [26] for confectionary, Cimini et al. [117] for pasta and Sieti et al. [130] for breakfast cereals, making this the most-noticed form of indirect effects. Direct connection to the (packaging-related) cause was again not in focus. Data were rather derived from reports instead of actual conducted studies for the respective food product under consideration [120,144].

In the reviewed studies, considerations of end-of-life (e.g., recycling, landfill, incineration) were varied. Some studies excluded the end-of-life phase altogether [116,122,123,125,127,128,131,133]. Some cited similar studies that excluded end-of-life due to many different scenarios that needed to be considered, making it difficult for standardization and comparison [116]. The remaining studies included end-of-life in some respect, either as end-of-life of packed food and/or end-of-life of the actual packaging solutions (often referenced as simply post-consumer waste, but also as the full packaging system, including primary, secondary and transport packaging). Though the end-of-life of packaging solutions was not often regarded as very significant in the results (as compared to other life cycle phases), commendably, some studies took a long and detailed look at the issue [117,120,121,129,130,132]. The inclusion and study of end-of-life scenarios are currently important, as with novel emerging products and materials, established waste management systems are continuously presented with new challenges to protect humans and the environment [145].

In terms of system boundaries, the picture is similar for EPDs. In principle, an attempt is made to cover the entire life cycle in three successive steps, namely upstream (e.g., raw material production, packaging and auxiliary material production), core (e.g., food production) and downstream (e.g., distribution up to shelf, primary packaging end-of-life). While most EPDs are limited to the named examples (e.g., EPD on crispbread [146]), others go beyond and include, for instance, domestic food losses or food preparation (e.g., cooking) (e.g., EPD on pasta [147]).

#### 3.2.2. Life Cycle Inventory

Table 2 lists the LCA studies reviewed and gives a comprehensive overview of the product (sub)categories, product names, the given packaging-related information, as well as the percentage of packaging-related GHG emissions.

##### Packaging

Focusing solely on packaging, in the category of confectionaries and the sub-category of cocoa and chocolate products, the primary level of packaging was in most cases aluminum foil [26,28,32,116,119,122,123,129] or combinations of aluminum foil with fiber-based packaging materials like paper [26,116,119,122,123,129] and board [26,32,129]. In some packages, additional packaging aids such as paper stickers were used [116], and information on finishing (e.g., print) [116] was given. Plastic packaging was less prominently represented. Found examples included chocolate-covered products (nuts) packaged in labelled plastic (low-density polyethylene (LDPE)) bags containing a modified atmosphere based on N_2_ [118], dark chocolate confectionary in a polyethylene terephthalate (PET) tray including a (corrugated) cardboard component, milk chocolate biscuit confectionary [26], as well as different chocolates [129] packaged in polypropylene (PP). Regarding the primary packaging concepts presented, product-typical solutions aimed at maintaining the product quality were given throughout. For example, the necessary barrier functions against light, oxygen, water vapor as well as aroma were met in almost all cases. In the cases where only plastic packaging (e.g., milk chocolate biscuit confectionary [26]; dark chocolate [129]) was mentioned and not further specified if a light barrier [150] in the form of a colored material or a secondary packaging level made of, e.g., cardboard was present, product quality and thus shelf-life may be potentially impaired [46]. The secondary packaging level of other products was exclusively fiber-based packaging, namely (corrugated) cardboard boxes [26,28,118], paper wrappers or boxes [116].

In the sub-category of other confectionaries, including breath-freshening micro-sweets, primary packaging concepts were similar to those given above and met product requirements which mainly covered protection from moisture uptake or loss [46]. Jelly and foam sweets [132], as well as milk-based confectionaries, were packaged in PP, while sugar confectionaries were packaged in aluminum foil and paper [26]. Secondary levels, where mentioned, were paper [26].

Cereals and cereal products, including the four sub-categories of whole, broken or flaked grain, flours and other milled products and starches, breakfast cereals as well as pasta, frequently used [46] plastic [117,124,125] and fiber-based [124,133] primary packaging concepts or a combination thereof [27,127,128,130]. All packaging concepts given aim to protect low-moisture or dried products (especially, e.g., breakfast cereals [27]) with low fat content from mainly water vapor, aroma, mechanical damage or oxidation [47]. In the case of ready-made wet porridge, a glass jar with an aluminum-plastic lid and alternatively a multilayer pouch with a cap was mentioned [130]. Secondary packaging levels were not thoroughly described, but if mentioned, they were mainly corrugated cardboard boxes [27,127,133] or cartons [117]. Additionally, high-density polyethylene (HDPE) [27], PP [127] or other unspecified plastic films [133] and labels [117] were named. One study even listed scotch tape used for closing cartons [117].

Comparing this with the EPDs found for this product group, one can see a strong overlap of packaging concepts. Flours and other milled products, for example, are likewise packaged in fiber-based solutions (paper bags) [151,152]. Additionally, bulk packaging (paper sacks, big plastic bags) is mentioned [153]. Breakfast cereals are packaged in plastic bags in paper box solutions [154], and pasta is packaged in either plastic [155,156,157,158,159,160,161,162,163,164,165,166,167], cardboard [156,157,168] or a combination thereof [147,157,158,169,170]. Additional packaging levels, where given, frequently included cardboard boxes, interlayers, pallets and plastic (stretch) films [147,154,155,158,159,160,161,162,165,166,167,168,169,170].

The shelf-life of bakery wares is significantly influenced by water exchange processes as well as interlinked structural changes, aroma uptake and (microbial) spoilage [46,47]. To limit this and prolong shelf-life, products in the sub-category of bread and rolls were primarily packaged in polyethylene (PE) bags [120], LDPE bags with (polystyrene (PS)) clips [11,134] or (wax-coated) paper bags [120]. Further, material combinations such as paper and polylactide (PLA) [131] or paper and PET [121] were used. Secondary packaging was (HDPE [121]) plastic boxes. In two sequential studies, it was stated that these were returnable [11,134].

The EPDs belonging to this product category, on the other hand, show only one packaging concept, namely that of a plastic bag with an associated clip. Additional packaging levels again include cardboard boxes and plastic films [171,172,173,174,175,176,177,178,179,180,181,182,183].

The sub-group of fine bakery wares showed a more diverse and elaborated packaging spectrum. While primary packaging for some biscuits was solely PP or a metallized PP film [30], others were packaged in multiple levels [29,31]. The latter may be due to higher product requirements in terms of quality. For example, cream fillings of biscuits as well as cakes [29,30] exhibit higher moisture and fat content and thus spoil more easily [46,47]. Additionally, elevated packaging [29,31] may be due to the fact that these products are more hedonistic than, e.g., cereal products such as breakfast cereals [184]. Secondary packaging in all given cases was cardboard/cardboard boxes [29,30].

The more diverse and elaborated packaging spectrum is also reflected in the EPDs. Here, different multilayer materials with or without paper are described. Additionally, different combinations of plastic or paper board trays, films, banderoles and/or boxes are given. Additional packaging layers are comparable to the above-mentioned ones [146,185,186,187,188,189,190,191,192,193,194,195,196,197,198,199,200,201,202,203,204,205,206,207,208,209,210,211,212,213,214,215,216,217].

Last but not least, the category of ready-to-eat savories and snacks, including potato-, cereal-, flour- or starch-based snacks using the example of crisps, were primarily packaged in a multilayer film made of oriented polypropylene (OPP) and metallized OPP [132], a common solution found in this category due to the superior gas and light barrier allowing stable product quality in terms of, e.g., crispness and lipid oxidation (rancidity) [46,47]. Processed nuts were packaged in LDPE bags with a label. Additionally, a modified atmosphere was applied [118] to protect the oxidation-sensitive products [46,47]. Secondary packaging (box, unspecified) was only given for the last-mentioned product [118].

Insofar as stated, tertiary packaging of all considered product (sub)categories was mainly represented by plastic materials such as (LDPE) (stretch-)films [28,29,30,117] and shrink-films [117] as well as (wooden) pallets [27,127,128]. Further materials described were cardboard/carton boxes [116], corrugated pallet layer pads [27] and labels [117]. In one case, an HDPE trolley was given [121]. Besides this, some authors even calculated consumer (plastic) bags in [28,30,121]. However, for the majority of products, no information on tertiary packaging levels was available.

Summing up, it can be seen from the reviewed studies taken together in Table 1 and Table 2 that predominantly plastic and aluminum packaging solutions were used in direct product contact. Further, it can be observed that packaging-specific information is not always given and that the detail of the same varies remarkably. Regarding the packaging levels, most authors give information on the primary packaging level, whereas secondary and especially tertiary levels are less frequently given [31,32,119,120,122,123,124,125,126,128,129,130,131,132,141]. In some cases, secondary and/or tertiary levels are even intentionally excluded [26,130,132]. Miah et al. [26], for example, justify not considering tertiary packaging (cut-off), for example, by the low weight percentage that comes from the tertiary packaging. Similarly, so do Sieti et al. [130]. Consequently, in many cases, only the primary packaging, and not the whole packaging system, is analyzed. This fact is also shown by Molina-Besch et al. [111]. Interestingly, different authors also seem to delineate packaging levels differently. For example, some authors include stretch films, which are often used to secure pallets [48], in secondary packaging [27,127,133], whereas others include them in tertiary packaging levels [28]. Additionally and interestingly, the EPDs under consideration distinguish between primary packaging and packaging for transport and do not go into detail about secondary/tertiary packaging levels (e.g., EPD on American sandwich [175]).

Furthermore, the level of detail of the information is deviating strongly. While some authors only mention the material, others include further information on, for instance, packaging containers (e.g., bag, tray, foil) [11,26,27,28,29,30,31,32,116,117,118,119,120,122,123,124,125,127,128,129,131,132,133,134], packaging aids (e.g., labels, adhesive tape, clips) [11,27,116,117,118,134], packaging weight [26,27,28,29,30,32,116,122,123,127,129,132,133], or dimensions [27,116], material composition (e.g., recycled content) [27,28,32,131], multilayer structure [27,30,132], usage of modified atmosphere packaging [118] or finishing processes such as printing [27,127]. EPDs usually reduce the information to the material used (e.g., EPD on crispbread [187]).

In some cases, information is directly included in the scientific paper, while in other cases, it is given as the supplementary material of the studies [26,28,29,30,32,117,118,123,127,129,130,134]. In addition, it is noticeable that packaging-specific information is often not given condensed at the beginning of the paper (e.g., materials and methods section, life cycle inventory) but spread over the text. Moreover, differences were also notable with regard to the data source. While some authors used primary data (e.g., specifications, information from companies), others used secondary data or based their calculations on assumptions. The most detailed information on packaging was found in the study by Cimini et al. [117].

##### Packaging End-of-Life

Regarding the packaging end-of-life, particularly waste management, country-specific scenarios are most frequently considered in studies where packaging (material) is mentioned and a cradle-to-grave approach is followed. This applies to, for example, rates of recycling, incineration or landfilling. For instance, Konstantas et al. [28] focused on chocolate production and consumption in the United Kingdom and included post-consumer waste management activities for the corrugated cardboard (recycling > incineration with energy recovery), aluminum (recycling > landfill) and plastic packaging (landfill > incineration with/without energy recovery) components. Additionally, efficiencies of the corrugated board and aluminum recycling processes were counted in. Further, authors who include disposal routes are, inter alia, Miah et al. [26] (United Kingdom), Bianchi et al. [129] and Cimini et al. [117] (Italy). Further, EPDs usually include primary packaging end-of-life (e.g., EPD on durum wheat semolina [151]).

Interestingly, most of the statements in the studies under review, as well as EPDs, are made based on, for example, reports on the national recycling rates of (packaging) materials (e.g., Cimini et al. [117,218]). The actual recyclability of the specific packaging solutions is, however, hardly addressed or analyzed in the reviewed studies [130,132]. This, however, is a knowledge field gaining importance and momentum in recent years [50], which is accompanied by different (e.g., design for recycling) guidelines [41], instruments and certificates (e.g., cyclos-HTP [219]). This becomes interesting, for example, in the case of very small packaging components or multilayer materials, for which the necessary sorting and recycling facilities often are not applied or even do not exist to date [52]. Accordingly, it is necessary to discuss whether the specified end-of-life scenarios are actually realistic and to what extent the results change.

##### Data Quality

It is well known that an LCA is only as reliable as the sources and dataset base it is built upon. Multiple sources and handbooks on LCA even state that data quality may largely determine LCA results [220]. In LCA, there are two main categories of data: primary and secondary. While primary data refers to actual data collected from sources of the investigated life cycle step (farmer, manufacturer, distributor etc.), secondary data refers to information from literature and databases. Quality thereof is, among other factors, determined by the recentness of the data and the model, geographical coverage, variability, representativeness and reproducibility [43,144]. The investigated studies took varied approaches to data quality issues. The sources for packaging LCA data were secondary in the majority of studies [11,26,27,28,29,30,32,116,118,120,122,125,128,129,130,134,141], whereas the remaining studies used primary and a mixture of primary and secondary data for packaging [31,117,121,123,126,127,131,132,133]. The actual sources of primary data were in-depth interviews and questionnaires with packaging producers, and for secondary data, the sources were the Ecoinvent and GaBi databases. Two of the studies were reviews that used published reports and results of other studies (published in journals), including their supplementary materials [11,141].

Espinoza-Orias et al. [120] and Jensen and Arlbjorn [134] took up the topic of data quality and usability of the like for sustainability assessment in the product category of bakery wares, specifically in the sub-category bread and rolls. The former authors even compared calculations between mainly primary and secondary sourced data (generic study). Other studies worth commenting on from the perspective of their attention to data quality are Usva et al. [126], who created a whole set of criteria for data quality and development and explained them fully in the text, as well as Cimini et al. [117], who used PAS2050 requirements for data quality, including geographic and time scope as well as technology references. This is in line with the CEN/TR 13910:2010 report on criteria and methodologies for LCA of packaging, which mentions the importance of giving special attention to time, geography and technology aspects within the data collection phase of LCAs [221].

#### 3.2.3. Life Cycle Impact Assessment

##### Impact Assessment Method and Impact Categories Used

As selected for, all of the examined studies assessed at least CO_2_ emissions/global warming potential (GWP)/carbon footprint of the food packaging systems [118,120,124,125,128,133,134,141]. In most cases, several other impact categories were also included. Examples are ozone depletion, fossil fuel depletion, terrestrial acidification, freshwater eutrophication, marine eutrophication and human toxicity [11,26,27,28,29,30,31,32,116,117,119,121,122,123,126,127,129,130,131,132]. The chosen impact categories depended on the used assessment method (e.g., ISO 14044 [67]) and the focus of the study in general. Using the above example of Espinoza-Orias et al. [120], two methodological approaches, namely PAS 2050 and ISO 14044 [67,108], were used. The former was used because it lays a focus on primary data, and the latter was used because the use of secondary data is allowed more. The aim was to compare the approaches and identify their influence on LCA results. It can be seen from this concrete example that the comparability of the studies is neither consistently given nor envisaged in this paper due to different scopes and applied assessment methods.

While carbon footprint is also covered by EPDs, other impact descriptive categories are, for instance, ecological footprint as well as water footprint (e.g., EPD on breakfast cereals [154]).

##### Sensitivity/Scenario Analysis

Of the present studies, only a few authors did not conduct a sensitivity/scenario analysis [122,124,125,126,128,132,141]. The others used this analysis to check for the robustness/generalizability of their results by alternating input data such as country of production [11,30,32,116,117,119,120,123,127,129,131,133,134]. Contrary to expectations, only a handful of studies included packaging in one or the other way in their sensitivity analysis [26,27,28,29,31,118,130]. For example, Volpe et al. [118] conducted an uncertainty and sensitivity analysis and concluded that abroad consumer markets and thus the final destination of (glass) packaging affect the LCA output (carbon footprint) significantly. However, the data for glass refers to nut spread cream packaged in a glass jar, which was excluded from the present review due to the product group exclusion reasons. Details for plastic bags used for the other products included in the present review were not given. Furthermore, Miah et al. [26] alternated packaging materials in an improvement analysis. Here, aluminum and PP were substituted with recycled material, paper with unbleached paper, and corrugated board with white lined board, while PET stayed unchanged. This led to “… a mix change in total environmental impact across all five confectionary products …” and, on average (across all confectionary products analyzed), an increase in GWP. Jeswani et al. [27], in the other case, exchanged some of the carton boxes with standalone HDPE bags in a hypothetical scenario, which resulted in a lowering of GWP. Additionally, Noya et al. [31] analyzed alternative waste management practices for packaging materials (increased recycling rates) with the result that the environmental burdens for the global process decreased (including climate change). Significance was, however, shown only for products with higher packaging requirements (plastic and cardboard). Last but not least, Konstantas et al. [29] focused on packaging losses (2 to 10%) in the manufacturing process and concluded that the results are not sensitive to packaging losses. Next to packaging, it can be mentioned that Miah et al. [26] and Noya et al. [31] also included food waste (reduction) in their analysis but did not interlink this with packaging (re)design. Surprisingly, although Williams and Wikström [11] had packaging embedded in their target, they did not conduct a corresponding sensitivity/scenario analysis.

#### 3.2.4. Interpretation

##### Environmental Impacts and Mitigation Measures

While Table 2 exhibits values of packaging-related CO_2_ emissions of different cereal and confectionary products on a single food item level, Table 1 provides an overview of product (sub) category-related emissions. As can be seen, single values range from 0.36 to 38.02% and in total, average packaging-related CO_2_ emissions account for 9.18%. Despite the fact that different studies are hardly comparable due to, for example, different aims, scope, system boundaries and input data, it becomes apparent that the average value lies clearly above the estimated general global values of about 5% by Crippa et al. [2] and Poore and Nemecek [3]. However, the values well reflect the wide possible variation previously found by, among others, Poore and Nemecek [3], Verghese et al. [6] and Heller et al. [7]. When going into detail about the different (sub)categories, interesting tendencies and hotspots can be found. These are discussed in the following paragraphs.

In the category of confectionary and, further, in the sub-categories of cocoa and chocolate products as well as other confectionaries, including breath-freshening micro-sweets, where average CO_2_ emissions (see Table 1) are 9.86 and 4.68%, respectively, the authors uni sono indicate that (raw)material sourcing is the main environmental impact driver. The provision and, in particular, the agricultural production of cocoa derivates, milk powder and sugar can be highlighted. This is also reflected by the environmental impacts of the respective products (Table 1). Boakye-Yiadom et al. [116] offer an illustrative example, where milk chocolate yielded significantly higher than dark or extra dark chocolate due to the high impact of the animal-derived food ingredients. Further, associated manufacturing processes and (fossil) energy consumption as well as (international) transport are ranked particularly high in the studies under review [26,28,32,116,118,119,123,129,132]. Further, reduction of (food)waste is mentioned as one way to cut carbon emissions [26,132]. In relation to packaging, behind the above-mentioned factors, significance has also been reported by different authors [26,28,116,118,119,129]. In this context, the main focus is on material choice [116,118,129]. In their work, Bianchi et al. [129] were able to show that a single PP layer is better than a combination of commonly used aluminum/fiber-based packaging solutions. Material (aluminum) substitution, if possible, is also on the agenda of Boakye-Yiadom et al. [116], who alternatively recommend using recycled or weight-reduced packaging solutions. Due to a lack of data, especially regarding thematic coverage, the studies [26,28,116,119] as well as Pérez-Neira et al. [123] do not go into detail about packaging but mention the importance of packaging optimization. Last but not least, collaboration with science and industry to develop packaging materials and solutions with lower impact were discussed by Miah et al. [26] and Boakye-Yiadom et al. [116].

Turning to cereals and cereal products, one can see that the average packaging-related CO_2_ emissions from whole, broken or flaked grain, flours and other milled products and starches, breakfast cereals as well as pasta are 1.25, 5.30, 19.68 and 7.24% (see Table 1), respectively. The significantly higher value for breakfast cereals is justified by the fact that wet porridge in a single-use glass jar was included in one study [130]. This is a packaging solution known for its high environmental impact, mainly due to very high process temperatures and, thus, energy needed in the production of the same [43]. Accordingly, the authors suggest replacing this with a lightweight plastic packaging solution (pouch), which exhibits 15.77 instead of 38.02% with regard to CO_2_ on a single product level [130]. A further change in material in the sub-category of breakfast cereals was proposed by Jeswani et al. [27], who found that replacing the well-known plastic bag and carton box combination for breakfast cereals with (standalone) plastic packaging (bags or pouches) could reduce carbon emissions. A possible preference for plastic packaging (PE bags) instead of paperboard boxes was also communicated by Cimini et al. [117] for dried pasta. The same authors also highlighted the correlation between high packaging density and the reduced packaging and transportation need for long pasta (e.g., spaghetti) in comparison with short pasta (e.g., spiral-shaped) due to the different shape and thus volume of pasta per functional unit. Furthermore, in the broader sense, relevant findings of packaging included the necessity to find the right trade-off between packaging function and environmental impact [141], to combine and prioritize actions [27,117], to engage relevant stakeholders (industry and government) to find best-practices and standards (e.g., packaging, types, mass reduction, recyclability) [130] and to intensify LCA applications and transparently communicate the results thereof (e.g., labelling) [124,141]. All in all, the packaging focus in this product category was less distinct than in the previous one, and the emphasis was mainly on the optimization of agricultural production and the provision of products [27,117,124,125,126,127,141], reformulation of recipes [128,130] and changing consumer habits. Here, for instance, the cooking of pasta [117,127], the consumption of cereal products with (cow’s) milk [27] or the use of ingredients of animal origin (egg, milk) [128,130] were related to higher impacts.

Since no EPDs for whole, broken or flaked grain are available to date [140], only comparisons of flours and other milled products and starches [151,152,153], breakfast cereals [154] and pasta [147,155,156,157,158,159,160,161,162,163,164,165,166,167,168,169,170] can be made at this point. Here, the average values are found to be 3.22, 12.37 and 8.56%, respectively. Although, as stated above, direct comparison is difficult, interestingly, a similar ranking can be identified. Therefore, flours and other milled products and starches score the lowest, while pasta and breakfast cereals, in ascending order, score higher. A possible explanation for this is the level of complexity of the packaging solutions. While milled, powdery products are densely packaged in simple bags, more volume-taking pasta is packaged in more stable and elaborately designed packaging solutions partly combining different materials. Breakfast cereals, in the present case, exhibit even higher packaging effort with a plastic bag and an additional cardboard box.

In the case of bakery wares, such as bread and rolls, as well as fine bakery wares, an average contribution of packaging to the CO_2_ emissions of 4.37 and 11.22% was found (Table 1). As expected, raw material (e.g., wheat, milk, palm oil, sugar) sourcing is the main environmental impact driver [29,30,31,120,121,131,134]. This is (not in strict chronological order) most often followed by processing and correlated energy use [29,30,131,134] as well as consumption (e.g., refrigeration, toasting) [120,134], although Svanes et al. [121] achieved a different result here. Further, waste at retail [121] and consumption level [120,121] as well as transport [30,31,120,131,134] and packaging are mentioned. The latter again played a less important role in other selected studies [29,30,120,121,131]. Of the packaging-related impacts, Konstantas et al. [30] named primary packaging as the most contributing factor. Several mitigation measures similar to the above product categories (e.g., efficient raw material sourcing) are given in the reviewed studies [11,29,30,31,120,121,131,134]. Regarding packaging, four main points were discussed by the authors, namely, portion size [120,121], packaging re-design [11,121] and light-weighting [29] as well as proper end-of-life management [31,134]. In the case of right-sizing portions, Espinoza-Orias et al. [120] as well as Svanes et al. [121] proposed that smaller sizes of bread (e.g., loafs) would reduce the amount of wasted bread (due to, e.g., spoilage) at the consumption stage but at the same time increase the need for packaging which, in the case of reduced food waste, still could lead to an environmental benefit–a finding that has already been shown in other contexts. Packaging re-design, on the other hand, included the substitution of a PET/paper packaging material with a material based on cellulose fibers and a perforated paper bag coated with PE on the inner side. While the former alteration allowed the bread to be kept fresher for one day, the latter solution allowed the product to be perceived as fresh even four days after production, which could lead to an environmental benefit since the impacts of producing the packaging alternatives are almost the same as with the packaging in comparison. The authors, who laid a strong focus on indirect packaging effects in their work, pointed out that further (large-scale) tests and the inclusion thereof in LCAs would be necessary to validate the results [121]. Studies on shelf-life extension strategies and waste prevention were also asked for by Williams and Wikström [11], who additionally highlighted that good product packaging should not encourage consumers to re-pack their products at home. This is a measure that could avoid unneeded extra packaging material. The latter also represents a recent research field where the understanding of consumer habits and social norms are focused, and food and packaging researchers are asked to more closely collaborate with social sciences and humanities [222]. Turning to the light-weighting of packaging, Konstantas et al. [29] calculated in their study on different cakes that a material reduction of 30% could lead to a significant drop in the GWP of cakes (except for whole cakes and cheesecakes). Food safety and shelf-life, however, must not be jeopardized as a result. The topic of end-of-life (improved waste management strategies and recycling rates [31,134]) was discussed by Jensen and Arlbjorn [134], who pointed out explicitly that hotspots should not only be identified on the basis of their impacts but also on the basis of their potential for change and that the awareness for possible burden shifting from one life cycle stage or impact category to another by just focusing on, for example, GWP values, should be kept at a high level.

Comparing the values found for the category of bakery wares and the sub-categories bread and rolls [171,172,173,174,175,176,177,178,179,180,181,182,183] as well as fine bakery wares [146,185,186,187,188,189,190,191,192,193,194,195,196,197,198,199,200,201,202,203,204,205,206,207,208,209,210,211,212,213,214,215,216,217] with the EPDs, values of 17.03 and 14.86% were found. In both cases, the values are higher than the ones from the studies under review. Possible causes for this may be, amongst others, the packaging material or the database used. The latter is frequently given to be mainly based on primary data. In the case of Italian bread (pagnotta), for example, it is stated that generic data contributes less than 10% to the calculation of environmental performance [182].

Lastly, in the category of ready-to-eat savories and snacks, which include potato-, cereal-, flour-, or starch-based snacks as well as processed nuts, the average contributions of packaging to the CO_2_ emissions were 8.14 and 20.10% (Table 1). Since these products were also covered by the already discussed research from Nilsson et al. [132] and Volpe et al. [118] in the product category of confectionary products, no further detail on packaging can be named at this point.

##### Significance of the Results

In their parallel (mainly primary/secondary data) studies on bakery wares (loaves of sliced bread), Espinoza-Orias et al. [120] conclude that data quality is key for not only the accurateness of the LCA results but also for honest sustainability communication. While secondary LCI data may be useful for rather uncomplicated (company) internal detection of hotspots or projections at the (inter)national level, high-quality primary data is needed for communication to consumers via, e.g., carbon labelling [138]. Similarly, Jensen and Arlbjorn [134] conclude that high-quality data is needed to achieve robust results.

In relation to impact assessment, Williams and Wikström [11] address food losses and food waste as well as packaging optimization in their conclusion. Here, they call for the inclusion of these indirect packaging impacts in food and packaging LCAs to examine how waste and, in consequence, negative environmental impacts can be diminished. Further, they highlight that legal texts should more strongly include the topic of food losses and food waste prevention by appropriate packaging solutions.

When talking not only about one impact category (e.g., GWP), a multi-criteria decision analysis (MCDA) as used, for example, by Miah et al. [26] can be helpful. This allows to compare different environmental impact categories together and to ease decision-making and benchmarking. Accordingly, MCDA is increasingly being used in LCA [223].

## 4. Improvement Strategies

As described at the outset, food systems are responsible for a large proportion of environmental impacts, especially GHG emissions, worldwide [2]. Increasing efficiency in food production and, above all, reducing food losses and waste can, therefore, directly contribute to lowering the global footprint [19,224]. In the last decade, the focus has therefore been on targeting, measuring and reducing GHG emissions. Along with that, efforts by different stakeholders have been conducted or started, and respective policies have been outlined [52,225]. Packaging is playing an increasingly important role in this context. While efforts initially focused on the reduction of the direct environmental impacts of packaging (e.g., material use), today, the focus is increasingly on the indirect impact (e.g., reduction of food waste), as it has been recognized that this has a potential leverage effect [13,34,52,110,226,227]. However, the actual inclusion of the indirect impact in research, development and innovation activities lags behind [111], as has also been shown by the present review. Accordingly, strategies for the acceleration of the implementation are needed. In this context, Wikström et al. [52] elaborated a research agenda including 5 packaging-related issues. These include: (i) quantitatively understanding packaging’s diverse functions and the influence on food losses and waste in the context of the (inter)national food system, (ii) more thoroughly understanding trade-offs between packaging and food losses and food waste, (iii) further improving representation thereof in LCA and (iv) designing processes and related methods as well as (v) setting stakeholder incentives such as profitable business models. To support this transition, the following text aims at aggregating possible points of action in the area of packaging, LCA and management beyond the topic of cereal and confectionary packaging.

### 4.1. Packaging

Starting with packaging, recommendations or suggestions found in this and other studies and texts can be very well set in the context of the existing Packaging Sustainability Framework with its four principles (effective, efficient, cyclic, safe) [42,43] (see also Table 3). This may act as a basis for future improvement regarding the reduction of the direct and indirect environmental impacts of food packaging. However, it must be clearly pointed out that there may be trade-offs and that verification of the respective product packaging system is essential [42,43].

Going into detail about the effectiveness of food packaging and analyzing the findings with regard to packaging that is fit for its purpose and, thus, is satisfactorily fulfilling its containment, protection, communication and convenience function [43,44,46,47], one can see that authors currently lay a focus on protection and convenience. Regarding protection, which is enabled by the often-overseen basis function of containment [46,47], the provision of an appropriate or prolonged shelf life is frequently mentioned [43,111,228,229,230]. In this context, the application of well-established and modern shelf-life extension practices [11], such as modified atmosphere packaging (MAP) [46,254] or active and intelligent packaging solutions (AIP) [46,47,255,256,257], can be named. Attention, however, should be paid to the possible over-engineering of packaging and not losing a holistic view of the packaging system. With regard to over-engineering, it may be reasonable to re-assess the actual product requirements and avoid unneeded packaging, as well as reduce packaging complexity or components, where possible. This can be supported by, for example, market research or research on consumption patterns [43]. With regard to a holistic view, the interlinkage between primary, secondary and tertiary packaging must be considered, since changes on one level may also necessitate changes on other levels. For instance, a reduced or less mechanically stable primary packaging (material) may induce the need to design the secondary or tertiary packaging to be more stable [43,111]. With respect to the convenience aspect of packaging, several authors take up the topic of developing packaging with a high consumer value or target group orientation. This includes, inter alia, packaging that is easy to open, reclosable or easy to empty and, in general, does not frustrate or even encourage consumers to re-pack products at home [10,11,43,46,111,223,258]. A point emphasized several times is also the right-sizing of portions to avoid food waste at the consumer level. This is a measure that, despite the increased packaging effort, can lead to a lower total environmental impact [111,120,121,228]. Next, the communication function of packaging, which has been somewhat overlooked by studies, could additionally play a significant role in food waste prevention in the future, as it can have a considerable influence on consumer behavior [12,33,259,260]. Examples of implementation would be easy to read and understand directions on how to store, prepare and use products or information on how to interpret best-before or consume-by dates, as well as how to dispose of the packaging [11,37,38,43].

Turning to the cluster of recommendations on efficiency, it can be seen that in the past, an emphasis was placed on this topic by many authors and that three hotspots are reoccurring. These are packaging itself, transport and energy. In the case of packaging, the majority of authors are looking for a sweet spot, a point where minimal packaging is used, but at the same time, the quality of the product is not affected. The same applies to product waste. In this context, however, it is necessary to mention that the impetus should come from the area of optimization rather than the pure minimization or elimination of packaging. This is reported to be a target-oriented approach to find a satisfactory balance between effort and impact [28,29,37,43,111,116,119,123,141,144,261]. Further emphasis in the scientific literature is laid on material choice or substitution as well as the (re)design of product-packaging systems. For example, some authors change traditional packaging concepts such as a bag in a box to a free-standing plastic bag or a glass jar to a plastic pouch. (Re)design examples, on the other hand, are packages exhibiting a perforation, a wide neck or that stand upside-down. All are attempts to increase the efficiency of product emptying and thus product waste, which may also be achieved by altering the product itself (e.g., rheology) [11,27,43,111,116,117,118,120,121,129,130]. Further, the use of, for example, concentrated products is discussed. This can also lead to reduced packaging effort. The latter is also of interest for transport efficiency. Here, packaging weight, avoidance of void volume and stack-ability stand in direct correlation to transport efforts (e.g., frequency) and thus impacts. The measures applied are, next to packaging weight, the packaging-to-product ratio, cube utilization (volume) and pallet utilization. Alternatively, and where possible, bulk shipping could also be a way to increase efficiency [43,111,141,261]. With respect to energy, choosing materials with low embodied energy and further increasing the efficiency of production processes and transport as well as detachment from fossil energy sources can be named. In addition to this, the consumer stage should not be underestimated. Here, a product-packaging system that does not need to be, for example, stored under refrigerated conditions or long-life packaging (e.g., aseptic packaging) may have advantages compared to other solutions [43,111].

As for the other areas, for cyclic packaging, different recommendations are given in the scientific literature. Clustering and (potentially) ranking them could be a valuable approach to link them with the well-established waste hierarchy, which is laid down by the EU Waste Framework Directive. Here, waste prevention as well as (preparing for) reuse are the most favored options. Behind this, recycling (including the technical and biological cycle) and energy recovery are mentioned. The least preferred option should be waste disposal through a landfill [36]. Through clustering, it becomes clear that most of the points discussed by different authors already focus on the upper part of the waste hierarchy. While the prevention of waste has already been discussed in the paragraphs above, reuse strategies given include reusable, returnable and refillable solutions not only at the primary packaging levels but also at the secondary or tertiary levels. Examples are (plastic) trays and crates, molded plastic containers for specialty products, (beer) kegs, intermediate bulk containers, roll cages or (wooden or plastic) pallets. It is important to consider that strategies may work in one case but not in another. Therefore, it is necessary to identify if the respective business-to-business or business-to-consumer case allows for such solutions. Situations where this often works well are those where short distribution distances, frequent deliveries, a small number of parties or company-owned vehicles are present. Therefore, a (custom) closed-loop system can be maintained [43,111]. Where reuse is not possible but waste is still generated, the collection, sorting, and forwarding of the respective waste fractions for recycling should be the main target [36,262]. To support this, the past years have shown a steep increase in guidelines focusing on design for recycling [37,39,41,43,261,263]. While these today focus mainly on mechanical recycling, chemical recycling may also be in focus in the upcoming years. A constant point of discussion is, however, the trade-off between lightweight multilayer materials exhibiting a small environmental footprint and their recyclability [50,264]. Next to designs for recycling, designs from recycling are increasingly the focus of science and industry since they are often associated with reduced primary material and energy consumption. The use includes materials of all categories, such as glass, metal, paper and board, as well as plastic. In the latter case, it must be, however, highlighted that at the moment, mainly recycled PET is used as primary food packaging material. Most approval processes for, e.g., PE and PP are still pending due to safety concerns [50,265]. Another trend in the past years is the increased production and use of bio-based and/or bio-degradable materials (e.g., polymers) [266]. The latter may be used in scenarios where entry into the environment is foreseeable. This could be either in the form of controlled (home or industrial) composting or in the form of uncontrolled littering. This could, in certain circumstances, reduce the amount of food waste going to landfill. While there is still a debate about the actual advantages (e.g., lower carbon footprint, material properties, bio-degradability) and disadvantages (e.g., agricultural impacts, competition with food production, end-of-life management, costs) of bio-plastics in different fields of applications [267], it is well agreed that all materials, regardless the material type, should be kept in the circle as long as possible and that proper end-of-life management is needed to reduce environmental impacts. Therefore, the transformation from a linear to a recycling and ultimately to a circular economy can be accelerated [35,36,262,268,269].

Last but not least, the area of safe packaging seems not to be in the forefront focus of the reviewed literature since the effects are mainly noticeable in other impact categories than GHG emissions. What can be said is, however, that the avoidance of hazardous substances (including GHG active substances) as well as cleaner production (e.g., avoidance of volatile organic components) can, next to ecological stewardship and litter reduction (e.g., small parts of packaging), support the transition towards a more sustainable future [35,37,43,44,261,268].

### 4.2. Life Cycle Assessment

In the past, a large number of LCAs were carried out in the food sector. It is clear that not every issue requires the inclusion of packaging. However, where packaging has been included in LCAs in one way or another, this often has not been sufficiently addressed [13,111]. The following paragraphs, therefore, aim to provide suggestions that show the potential to improve the quality of future studies and the validity of packaging-related conclusions drawn from them. To structure this, the multi-step approach based on ISO 14040 and 14044, (i) goal and scope, (ii) life cycle inventory, (iii) life cycle impact assessment and (iv) interpretation, is used again for this purpose [66,67] (see also Table 4).

Starting with the goal and scope of a packaging-related LCA, it has to be stressed that the holistic representation of the entire food packaging system is a prerequisite for all further steps. This means that packaging relevant points beyond production and waste management have to be included. These are, for example, indirect effects such as food waste or transport efficiency along the supply chain. Further, all packaging levels, from primary to tertiary packaging, should be considered, and awareness of their interrelationship should be given. This is relevant, for example, in comparative studies where different packaging variants are included [43,111,221].

Another issue that is worth addressing is the increasingly important concept of the Circular Economy. A new legislative initiative undertaken by the European Commission in adopting the Circular Economy Action Plan in 2015 had a significant impact on the field of packaging. This initiative led to changes in existing directives and the imposition of stricter rules as well as the introduction of the Product Environmental Footprint (PEF) circularity formula [270].

Further, the CEN/TR 13910:2010 report on criteria and methodologies for LCAs of packaging also mentions the importance of time, geography and technology aspects within the goal and scope definition as well as data collection phases of LCA. These time and technology aspects are important due to the characteristically short life cycle of packaging (e.g., design changes). The geographical aspect considers different supply chains across several countries and continents [221].

Building upon this sharpened approach, it is further necessary to increase efforts in the area of life cycle inventory to achieve meaningful results. First and foremost, data quality can be mentioned here [43,120,134]. Although it is well-known that data gathering can be quite resource-intensive (e.g., time, budget), ideally, primary data (e.g., directly (on-site) collected data) should be used. However, if not otherwise possible, secondary data (e.g., database, reports, statistics) may also be taken. Furthermore, in some cases, assumptions may be necessary [43,52,120,134]. With secondary data selection, there is also another issue. LCA software very often comes bundled with specific databases, and there is evidence that the choice of software used for environmental analysis can affect the relative comparisons between differing package system options and, therefore, the decisions that will be made. This effect is magnified by the natural inclination of the user to employ data sets that are “convenient” when using specific software packages [276]. Regardless of the source, however, it is helpful to present the information in the studies themselves or in the appendix in a transparent and bundled manner in order to promote the progress of the research field as well as comparability. This is a point that is increasingly requested by different stakeholders and encouraged by scientific journals on LCA such as The International Journal of Life Cycle Assessment and Environmental Impact Assessment Review [220,277]. Moreover, care should be taken to use widely accepted definitions (e.g., ISO standards) to avoid the misinterpretation of, for example, packaging levels [51].

In relation to primary, secondary and tertiary packaging, it is advisable to collect information that exceeds the one on the base material used. This refers to information on the packaging material (e.g., exact material, size, additives, barrier, color, print), packaging aids (e.g., closure, liner, gasket, valve) and decorations (e.g., labels, adhesives, decoration, size) [41] as well as any other relevant points such as modified atmosphere packaging (MAP) [46,254] or active and intelligent packaging (AIP) [46,47,255,256,257]. Although, at first glance, it may seem a bit far-fetched, addressing these points helps to assess the actual recyclability of a packaging solution in a target market or region (e.g., by using (inter)national guidelines) and potentially point out improvement possibilities [39,263]. Looking at the markets in more detail, it should be noted that some (federal) states have different collection, sorting and recycling practices, which means that recovery rates may differ in some cases from the average values for a country [278]. Accordingly, more focus should be placed on these currently rather underrepresented points to further increase the validity of LCA results.

Further, more attention should be paid to food and packaging waste generated at different supply chain stages (e.g., production waste, loss during transport and retail) and where the remainder of this waste is. Especially in efficiency-driven countries, data up to retail is often available. At the consumer level, however, the data situation is often less satisfactory. Therefore, more attention should be paid to better understanding consumer behavior and attitudes in the future. Points of interest could be consumers’ preference for food/packaging, un/re-packing habits, storage and use of products, food waste as well as engagement in separation and disposal of packaging and preference for, e.g., bio-based and biodegradable/compostable packaging materials [56,111].

Turning to the LCIA, it can be reiterated that existing (e.g., ISO) and recently developed standards (e.g., PEF) provide a solid basis for the calculation of environmental impacts [66,67,102,103]. In the context of these, sensitivity or scenario analyses are mentioned, as they are a method to check for the validity of results or to describe possible variations/situations [66,67]. Applying this supports the authors if, for instance, different assumptions have to be made or the importance of different packaging attributes is to be tested [52,111]. A possible approach in relation to, for example, food waste originating from different packaging solutions would be the following: (i) examination of the situation (e.g., amount, reason) and gathering of supporting primary (e.g., experiments) or secondary data (e.g., literature), (ii) identification, definition and evaluation (e.g., experiments) of influencing packaging attributes, (iii) scenario development (e.g., alteration of packaging size) and evaluation as well as (iv) calculation and interpretation of results [52] based on [12,13,275].

Last but not least, interpretation of results has the potential to be improved in future LCAs. Depending on whether the respective study has a packaging focus (*packaging* LCA) or not (*food* LCA), different recommendations can be found in the literature. For *packaging* LCAs, awareness about limitations (even implicit ones) of the conducted study as well as transparent reflection thereof in the corresponding discussion can be highlighted [43,52,111]. This should include, once more, currently underrepresented points such as interdependencies of packaging levels, consumers or waste-related issues [52,111,221]. Furthermore, trade-offs and possible burden-shifting can be addressed using, for example, single-score values or multi-criteria decision analysis (MCDA) [31,134]. Where such critical discourse is, e.g., due to space limitation, not possible, giving recommendations or directions for packaging (re)design should therefore be refrained from. On the contrary, it would be more beneficial to underline the need for further research. The latter also applies to *food* LCAs [111].

### 4.3. Management

When it comes to promoting sustainable food packaging systems, different challenges and opportunities exist. The challenges include, for example, established economic systems that are traditionally strongly oriented toward growth and profit and are slow to implement necessary changes. In addition, there is often a need for improved holistic sustainability awareness, networking and exchange with the economic environment. This finds reflection until the single company and department level [43,52].

In order to more easily overcome the activation energy required for a change, various catalytic measures can be adopted on different levels (see also Table 5). At a meta or policy level, which rather reflects a top-down approach, incentives [52,111] such as corresponding legal frameworks, facilitation for exemplary companies [15,268,279], as well as support or funding for research, development and innovation can be named [222,280]. This motivates companies along the food supply chain to develop new business models in which saving resources and reducing or avoiding food losses and food waste are valued and gains and risks are shared equally [52]. Further impetus provides strong engagement and the cross-linking of relevant stakeholders (e.g., industry, government [130]) to promote best practices (e.g., recyclable packaging), standards, as well as an open (science) approach [274,281,282]. Education offensives at different levels are also seen as helpful. Therefore, for example, more and more schools and universities include packaging in their curricula [283].

Next to this, the bottom-up approach also bears huge innovation potential. In particular, a lot can be expected from companies that, with reference to the sustainability phase model, have already left the phases of rejection, non-responsiveness, compliance and efficiency behind them and are already operating at the levels of strategic proactivity and a sustaining corporation [43,285,287]. As above, the cooperative approach should be emphasized here. For instance, science and industry can collaborate to develop improved food and packaging solutions, or communication along the supply chain can promote overall sustainability and avoid double efforts [26,43,116,130].

At the company level, the management of sustainable packaging development should target the identification of environmental hotspots and potentials for change (see also Section 4.2) as well as combining and prioritizing actions (see also Section 4.1) [27,117]. Here, it is especially important that supposedly more sustainable packaging approaches or solutions are also tested extensively (e.g., packaging performance, product quality, shelf life and waste, consumer attitudes and handling, environmental impact) in order to ultimately bring a product onto the market that is successful in all dimensions [43,46,47,48,70]. In times like these, when different consumers and other stakeholders are becoming increasingly aware of the sustainability of food packaging [74], it is vital to communicate the developments made in a transparent manner and provide factual information about the sustainability aspects of packaging. Explicit (e.g., text, labels, certificates) and implicit (e.g., pictures and graphics, colors, haptics, font, shape) communication thereby can take place through a variety of channels [56]. This can include, for example, on the packaging itself, but also on websites or various other advertising channels [121,138,140]. Whichever way is used to communicate, it is particularly important that there is no misleading or greenwashing [124,138,141,259,286] in this context, which is picked up in a recent initiative on substantiating green claims by the European Union [255,288,289].

## 5. Conclusions

In the past, it has been shown that packaging can have positive environmental effects, especially when it protects resource-intensive food products and thus prevents losses and waste of the same. This is an essential point when it comes to reducing GHG emissions associated with the global food supply chain. In the present review with a focus on LCA studies, it was shown that the average contribution of packaging to the overall footprint of the product packaging system is 9.18% for the product group of cereals and confectionery, which has not been the explicit focus of scientific literature to date. This value is approximately twice as high as the estimated value for global GHG emissions for packaging but fits in well with previous dimensions for packaging of various food groups, which range from a few percent to more than one-third. In this context, however, it must be emphatically pointed out that direct comparisons in this area are not permissible or are difficult to carry out, as the studies differ greatly in some cases. The results can therefore be seen more as a size estimate.

In addition, the present review provided valuable information about the type and quality with which packaging has been included in analyses so far. In particular, it showed that packaging was often not in focus, and if it was, it was often not sufficiently included at all levels (primary, secondary and tertiary). It also showed that mainly direct (e.g., material) and not indirect impacts (e.g., food waste, transport efficiency) were considered and that data quality and presentation could be improved.

Based on these evaluations and including further literature, recommendations for the sustainable design of food packaging, its analysis by means of LCA and innovation-supporting management could be given. In the area of packaging, it can be particularly emphasized that packaging must be designed to be effective, efficient, recyclable and safe, and that interrelationships between the individual packaging levels must always be considered. With LCA, on the other hand, it is necessary not to lose sight of packaging from the beginning, including the definition of the goal and the scope, through the LCI process over LCIA to the interpretation and issue of recommendations. In addition, to obtain accurate results, primary data should be used whenever possible, while secondary data are recommended for a rough estimate of influences. LCA practitioners should also refrain from issuing packaging-related recommendations if these have not previously been sufficiently included in the studies. In this case, the reference to the need for further studies is more appropriate. Last but not least, the management-related part dealt with how innovation can be fueled at different levels and showed that collaboration as well as transparent and honest communication of sustainability aspects within the supply chain and towards the consumer is a key instrument for realizing sustainability at all levels.

Against this background, the authors see considerable research and development potential in the areas of better coverage of the cereal and confectionary product group, optimization of packaging and evaluation of the actual influence of the same, the meaningful design of LCAs, the demonstration of indirect packaging effects along the supply chain, new business models and models for cooperation as well as communication of sustainability aspects.

## Figures and Tables

**Table 1:** Reviewed cereal and confectionary life cycle assessment (LCA) studies (*n* = 28).

Category	Sub-Category	LCAs * *n* = 28		Products *n* = 108		Greenhouse Gas Emissions		
		*n*	%	*n*	%	Food-Packaging System [kg CO_2_eq]	Packaging [kg CO_2_eq]	Packaging (%)
Confectionary	Cocoa and chocolate products	9	32	41	38	3.28	0.25	9.86
	Other confectionary including breath-freshening micro-sweets	2	7	4	4	2.80	0.16	4.68
Cereals and cereal products	Whole, broken or flaked grain	2	7	9	8	12.53	0.14	1.25
	Flours and other milled products and starches	2	7	3	3	0.65	0.04	5.30
	Breakfast cereals	2	7	4	4	0.87	0.15	19.68
	Pasta	4	14	10	9	1.33	0.10	7.24
Bakery wares	Bread and rolls	5	18	20	19	1.03	0.04	4.37
	Fine bakery wares	3	11	12	11	1.93	0.04	11.22
Ready-to-eat savories and snacks	Potato-, cereal-, flour- or starch-based snacks	1	4	1	1	0.43	0.04	8.14
	Processed nuts	1	4	4	4	1.87	0.33	20.10
Overall (average)						2.67	0.13	9.18

* Some LCA studies covered more than one (sub)category. Therefore, given numbers do not sum up to *n* = 28 or 100%.

**Table 2:** Reviewed cereal and confectionary life cycle assessment (LCA) studies: information on packaging and its percentage share of total greenhouse gas (GHG) emissions.

Category	Sub-Category	Product	Primary Packaging Level	Secondary Packaging Level	Tertiary Packaging Level	GHG [%]	Ref.
Confectionery	Cocoa and chocolate products	Chocolate-covered hazelnut	Modified atmosphere in LDPE bag, label	Box	-	17.80	[118]
		Chocolate-covered almond	Modified atmosphere in LDPE bag, label	Box	-	6.00	
		Dark chocolate	Aluminum foil, cardboard	-	-	13.02	[32]
		Chocolate (100%)	Aluminum foil, paper	-	-	8.56	[122]
		Malty chocolates (in bags)	Aluminum foil	Corrugated cardboard boxes	LDPE stretch-film, LDPE consumer plastic bags	13.00	[28]
		Chocolate-coated wafers (contlines)	Aluminum foil	Corrugated cardboard boxes	LDPE stretch-film, LDPE consumer plastic bags	8.00	
		Milk chocolate (molded)	Aluminum foil	Corrugated cardboard boxes	LDPE stretch-film, LDPE consumer plastic bags	6.00	
		Milk chocolate	Aluminum foil, paper	-	-	6.94	[119]
		Dark chocolate				11.90	
		White chocolate				6.10	
		Chocolate with sultanas				10.42	
		Milk chocolate confectionary	Aluminum foil	Corrugated board box	Not considered	2.27	[26]
		Dark chocolate confectionary	PET tray, corrugated cardboard component	Corrugated board box		5.18	
		Milk chocolate biscuit confectionary	PP film	Corrugated board box		3.00	
		Dark chocolate	PP	-	-	4.71	[129]
		Dark chocolate	Aluminum foil, fiber-based layer (cardboard)			24.87	
		Dark chocolate	Aluminum foil, fiber-based layer (Kraft paper)			18.82	
		Milk chocolate	PP	-	-	2.20	
		Milk chocolate	Aluminum foil, fiber-based layer (cardboard)			11.65	
		Milk chocolate	Aluminum foil, fiber-based layer (Kraft paper)			8.82	
		White chocolate	PP	-	-	2.26	
		White chocolate	Aluminum foil, fiber-based layer (cardboard)			11.94	
		White chocolate	Aluminum foil, fiber-based layer (Kraft paper)			9.04	
		Extra dark chocolate, 65 g strip	Paper covered Aluminum foil, paper sticker	Paper box	Cardboard/carton box	23.64	[116]
		Dark chocolate, 65 g strip				23.35	
		Milk chocolate, 65 g strip				9.31	
		Flavored milk chocolate, 65 g strip				9.26	
		Extra dark chocolate, 100 g bar	Aluminum foil)	Printed paper wrapper	Cardboard/carton box	12.12	
		Dark chocolate, 100 g bar				11.98	
		Milk chocolate, 100 g bar				4.77	
		Flavored milk chocolate, 100 g bar				4.75	
		Extra dark chocolate, 300 g pouch	Paper covered aluminum foil, paper sticker	Paper box	Cardboard/carton box	13.94	
		Dark chocolate, 300 g pouch				13.77	
		Milk chocolate, 300 g pouch				5.49	
		Flavored milk chocolate, 300 g pouch				5.46	
		Conventional monoculture chocolate (min. transport)	Aluminum foil, paper	-	-	8.71	[123] based on [32,122]
		Conventional agroforestry chocolate, (min. transport)				11.84	
		Organic agroforestry chocolate, (min. transport)				13.24	
		Conventional monoculture chocolate, (max. transport)				5.79	
		Conventional agroforestry chocolate, (max. transport)				7.03	
		Organic agroforestry chocolate, (max. transport)				7.50	
	Other confectionaries, including breath-freshening micro-sweets	Jelly sweets	PP bags	Not included	Not included	8.75	[132]
		Foam sweets	PP container			1.88	
		Sugar confectionary	Aluminum foil, paper	Corrugated board box	Not considered	5.26	[26]
		Milk-based confectionary	PP film	Corrugated board box		2.85	
Cereals and cereal products	Whole, broken or flaked grain	Rice (IT)	Plastic bag	-	-	1,95	[124]
		Rice organic (IT)				1.33	
		Rice (US)	Cardboard box			0.36	
		Rice parboiled (US)				0.91	
		Rice upland (CH)				1.82	
		Minimal tillage white rice	LDPE bags	-	-	1.46	[125]
		Minimal tillage brown rice				1.82	
		Organic cultivation white rice				0.62	
		Organic cultivation brown rice				1.02	
	Flours and other milled products and starches	Oatmeal	-	-	-	6.02	[126]
		Potato flour				7.69	
		Wheat flour	-	-	-	2.17	[141] based on [148]
	Breakfast cereals	Breakfast cereals	Printed board folding-box, HDPE bag/liner	Corrugated-board box, HDPE stretch film/wrap	Corrugated pallet layer pads, Wooden pallet	15.00	[27]
		Dry ready-made porridge	LDPE bag, cardboard box (“bag in box”)	Not considered	Not considered	9.93	[130]
		Wet ready-made porridge	Glass jar, cab (aluminum and plastics)			38.02	
		Wet ready-made porridge (scenario)	Pouch, cap			15.77	
	Pasta	Dried short pasta 0.5 kg	Re-closeable PP bag	Carton, adhesive label, scotch tape	Stretch and shrink film, label, EPAL wood pallet, different layers of cartons	5.90	[117]
		Dried long pasta 0.5 kg	Re-closeable PP bag			3.40	
		Dried short pasta 0.5 kg	Paperboard box			13.90	
		Dried long pasta 0.5 kg	Paperboard box			9.40	
		Dried short pasta 3 kg	PE bag			8.20	
		Dried long pasta 3 kg	PE bag			3.10	
		Pasta	Paper	Cardboard paper, plastic film	Corrugated board	1.00	[133]
		Pasta (wheat, 0% straw)	Low-density PET film, cardboard box, printing	Corrugated board, PP film	Pallet	10.00	[127]
		Pasta (wheat, 80% straw)				10.20	
		Pasta (egg)	-	-	Pallet	7.26	[128] based on [149]
Bakery wares	Bread and rolls	White bread (medium slices, 40 g)	PE bag	-	-	1.61	[120]
		Wholemeal bread (medium slices, 40 g)				1.73	
		White bread (thick slices, 57.5 g)				1.67	
		Whole meal bread (thick slices, 57.5 g)				1.80	
		White bread, medium slices (generic study)				2.73	
		Wholemeal bread, medium slices (generic study)				2.91	
		Brown bread, medium slices				2.84	
		White bread, thick slices (generic study)				2.86	
		Wholemeal bread, thick slices (generic study)				3.07	
		Brown bread, thick slices (generic study)				2.99	
		White bread (medium slices, 40 g) (generic study)				5.31	
		Wholemeal bread (medium slices, 40 g) (generic study)	Wax coated paper bag			5.66	
		Brown bread, medium slices (generic study)				5.51	
		White bread (thick slices, 57.5 g) (generic study)				5.56	
		Whole meal bread (thick slices, 57.5 g) (generic study)				5.95	
		Brown bread, thick slices (generic study)				5.80	
		Bread (wheat)	Paper bag (paper and polylactide)	-	-	11.58	[131]
		Rye bread	LDPE bag, plastic clip	Returnable plastic box	-	6.10	[134] based on [11]
		Bread	PET and paper	HDPE box	HDPE trolley, extra packaging used by consumers	7.07	[121]
		Bread	LDPE bag, PS clip	Returnable plastic box	-	4.59	[11]
	Fine bakery wares	Biscuits	Tray, wrap, cardboard case, plastic film	-	-	17.62	[31]
		Crackers	PP film	Cardboard box	LDPE film, LDPE shopping bag	7.00	[30]
		Low fat/sugar biscuits	PP film		LDPE film, LDPE shopping bag	6.00	
		Semi-sweet biscuits	PP film		LDPE film, LDPE shopping bag	6.00	
		Chocolate-coated biscuits	PP film		LDPE film, LDPE shopping bag	4.00	
		Sandwich (Chocolate cream) biscuits	Metallized (aluminum) PP film	Cardboard box	LDPE film, LDPE shopping bag	8.00	
		Sandwich (vanilla cream) biscuits				7.00	
		Whole cakes	PP, cardboard folding box	Cardboard	LDPE wrap, consumer shopping bags	7.00	
		Cake slices	Cardboard folding box, LDPE	Cardboard	LDPE wrap, consumer shopping bags	19.00	[29]
		Apple pie	Cardboard folding box, LDPE, aluminum foil	Cardboard	LDPE wrap, consumer shopping bags	24.00	
		Cupcakes	Cardboard folding box, LDPE, paper	Cardboard	LDPE wrap, consumer shopping bags	24.00	
		Cheesecake	PP, cardboard folding box, LDPE	Cardboard	LDPE wrap, consumer shopping bags	5.00	
Ready-to-eat savories and snacks	Potato-, cereal-, flour- or starch-based snacks	Crisps	OPP and (aluminum) metallized OPP	Not included	Not included	8.14	[132]
	Processed nuts	Pistachio	Modified atmosphere in LDPE bag, label	Box	-	12.80	[118]
		Almond				12.90	
		Hazelnut				29.80	
		Peanut				24.90	

**Table 3:** Recommendations for improving the sustainability of food packaging based on the structure given by [36,46].

Sustainable Packaging Principle	Recommendation	Reference
Effective	Usage of packaging fit for purpose	[43,44,46]
	Provision of appropriate shelf-life	[43,111] based on [228,229,230]
	Employment of shelf-life extension strategies	[11,231]
	Avoidance of over-engineering	[43]
	Holistically integrate primary, secondary and tertiary packaging levels	[43]
	Provide packaging with high consumer value	[10,11,43,111] based on [229]
	Target-group oriented packaging with consumer value	[10,11,43,111] based on [229]
	Right-sized portions	[111,120,121] based on [120,228,229]
	Provide clear and understandable communication	[11,37,43]
Efficient	Optimize packaging with regard to function and environmental impact	[26,28,29,37,43,111,116,119,123,141] based on [27,232,233,234,235,236,237,238,239,240,241,242,243,244,245]
	Rethink material choice and packaging design	[10,27,43,111,116,117,118,121,129,130] based on [27,120,233,235,236,238,240,244,246,247,248,249,250,251]
	Increase transport efficiency	[43,111,141] based on [232,237,244]
	Decrease energy demand along the supply chain (e.g., process and transport)	[43,111] based on [243]
	Focus on renewable resources (materials and energy)	
Cyclic	Avoid unneeded packaging	[111] based on [252]
	Prevent and reduce food and packaging waste along the supply chain	[26,43,111,132] based on [242];
	Use reusable, returnable or refillable (primary, secondary, tertiary) packaging solutions	[43,111] based on [240,246,252,253]
	Design packaging *for* recycling	[35,37,39,41,43]
	Design packaging *from* recycling	[37,43,111,116] based on [230,231,244,248,249]
	Use bio-based and/or bio-degradable materials	[37,43,44,111]
	Assure proper end-of-life management	[31,43,134]
	Promote a circular economy	[35,36],
Safe	Focus clean production	[35,37,43,44]
	Install ecological stewardship	[37,43]
	Reduce possibility for litter formation	[43]

**Table 4:** Recommendations for improving food packaging life cycle assessments (LCAs) based on the structure given by [66,67].

Life Cycle Assessment Stage	Recommendation	Reference
Goal and scope	Holistic representation of the food packaging system	[43,111]
	Inclusion of all packaging levels	[43,111]
	Inclusion of direct and indirect packaging effects	[43,52,111]
	Awareness of interrelation	[43,111]
	Integration of Circular Economy principles within the goal and scope of food packaging LCAs	[270,271,272]
	Special attention to time, geography and technology aspects	[130,221,273]
Life cycle inventory	Focus on appropriate and reasonable high-quality data and software	[43,52,120,134,144]
	Provision of data transparency and consistency	[274]
	Usage of common language (definitions)	[51]
	Inclusion of details on packaging	[41]
	Inclusion of actual packaging recyclability and recycling quotas	[39,41]
	Inclusion of food and packaging waste	[111]
	Inclusion of consumer attitudes and behavior	[111]
Life cycle impact assessment	Use and build upon standards	[66,67,102]
	Include sensitivity or scenario analyses	[52,66,67,111] based on [12,13,275]
Interpretation	Discuss limitations	[43,52,111]
	Address trade-offs and burden-shifting	[31,134]
	Use multi-criteria decision analysis (MCDA)	[31,134]
	Only give sufficiently substantiated recommendations	[52,138]

**Table 5:** Recommendations for management-related activities to promote sustainable packaging.

Recommendation	Reference
Give incentives	[52]
Develop new business models	[52]
Engage and connect stakeholders	[130]
Follow an open (science) approach and promote best practices and standards	[274,284]
Promote education	[283]
Develop companies to sustaining corporations	[43,285]
Strengthen collaboration and communication	[26,116,130]
Avoid double efforts	[26,116,130]
Identification of environmental hotspots and potentials for change	[27,117]
Combine and prioritize actions	[27,117]
Extensively test (re)designed packaging solutions	[43,46,47,48]
Communicate sustainability aspects transparently and provide evidence	[121,138]
Avoid misleading or greenwashing	[124,141,286]
